# Hyperscanning Alone Cannot Prove Causality. Multibrain Stimulation Can

**DOI:** 10.1016/j.tics.2020.11.003

**Published:** 2021-02

**Authors:** Giacomo Novembre, Gian Domenico Iannetti

**Affiliations:** 1 Neuroscience and Behaviour Laboratory, Italian Institute of Technology, Rome, Italy; 2 Department of Neuroscience, Physiology and Pharmacology, University College London, UK

**Keywords:** social interaction, hyperscanning, causality, multibrain stimulation, joint action

## Abstract

Brains that work together, couple together through interbrain synchrony. Does interbrain synchrony causally facilitate social interaction? This question cannot be answered by simply recording from multiple brains (hyperscanning). It instead requires causal protocols entailing their simultaneous stimulation (multibrain stimulation). We highlight promising findings and future horizons of this nascent field.

## Hyperscanning and Social Interaction

The simultaneous recording from multiple brains – hyperscanning – has led to many reports of interbrain synchrony among socially interacting individuals. Such states of synchrony appear to facilitate social behaviors such as interpersonal coordination, cooperation and communication [1,2]. This phenomenon has deep ontogenetic and phylogenetic roots. It is observable in early mother–child interactions [1], and it is well conserved across a number of social species [2].

Social interactions, by definition, entail information transfer between two or more individuals, typically through acoustic or visual signals (for the sake of conciseness, from here onwards we only refer to two individuals). It follows that nearly all hyperscanning studies record from multiple brains while they are exposed to similar sensory input. This consideration raises a fundamental question concerning the nature of interbrain synchrony. Can it be conceptualized as a neural mechanism that causally facilitates social interaction, or is it an epiphenomenon that by itself has no direct effect on social interaction but simply emerges as a consequence of two brains encoding a similar sensory environment?

## Interbrain Synchrony: Mechanism or Epiphenomenon?

The mechanistic perspective postulates that two individuals achieve interbrain synchrony to better function in a social context. This could be considered a generalization of the widely accepted principle that two distinct areas of the same brain communicate through coherent neural activity [3], although applied across two separate individuals. That is, two areas, each in a different brain, would optimize social behavior when their rhythms are synchronized. Indeed, as the brain samples information from the environment rhythmically rather than continuously, synchronizing two neural rhythms across two brains could effectively facilitate interpersonal information flow [4].

Alternatively, the epiphenomenal perspective postulates that interbrain synchrony arises as a mere consequence of the fact that two individuals share the same sensory environment or perform the same task. Indeed, two brains receiving the same input (or performing the same movements) would simply display similar neural responses at comparable latencies. This would in turn lead to spurious synchrony, purely epiphenomenal in nature and not having a role in causally determining or facilitating the motor output underlying the social behavior. Although this perspective has been discussed as plausible in the most recent reviews of the field [1,2], conclusive empirical evidence is lacking.

## The Importance of Causal Evidence

How can we distinguish between the mechanistic and epiphenomenal interpretations of interbrain synchrony? Here, we argue that hyperscanning alone, while providing important correlational evidence, cannot produce any substantial leap towards addressing this fundamental question. Obtaining causal evidence is therefore necessary (see Box 1 for a discussion on how to infer causality in neuroscience).

To illustrate the difference between correlational and causal evidence, imagine a scientist who needs to reverse engineer an electronic device. Observing that a certain pattern of current flow (A) within a circuit is always followed by a light turning on (B) implies that A and B are somehow related to one another. Yet, this alone does not imply that A is causing B. To prove a causal relationship, one needs to manipulate the pattern (A), and examine the effects on the light (B).

**Figure 1:**
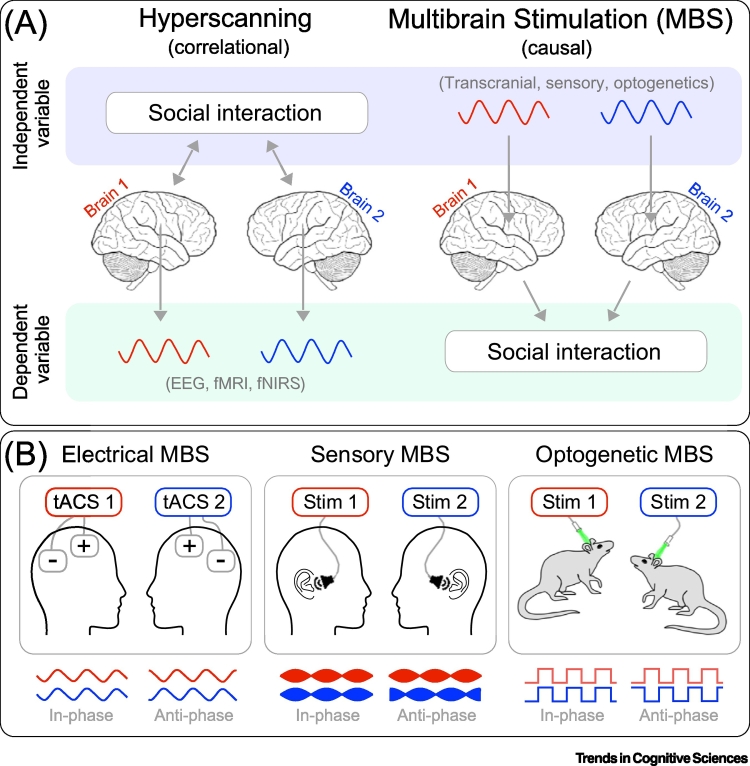
MBS: Theory and Practice. (A) Hyperscanning versus MBS. In Hyperscanning, inter-brain synchrony is measured during social interaction (e.g., with electroencephalography, functional magnetic resonance imaging, or functional near-infrared spectroscopy). In MBS, interbrain synchrony is exogenously modulated (e.g., with transcranial stimulation, sensory stimulation, or optogenetics), to test its causal effects on social interaction. Causal effects of interbrain synchrony cannot be proved with hyperscanning, due to its intrinsic correlational nature. (B) Present and future applications of MBS. MBS can be achieved using noninvasive neurostimulation (e.g., tACS in humans; left), rhythmic sensory stimulation (e.g., acoustic stimulation causing neural entrainment outlasting the stimulation offset; middle), or invasive neurostimulation (e.g., optogenetics in rats; right). For illustrative purposes, we depict only in-phase and anti-phase relationships between the stimuli delivered to two brains, although several other stimulus properties could be manipulated. Abbreviations: MBS, multibrain stimulation.

Along the same line, scientists should not solely report correlations between interbrain synchrony (assessed by hyperscanning) and social behavior. They should also exogenously manipulate interbrain synchrony and carefully assess its causal effects on social interaction (Figure 1A). Such exogenous manipulation of interbrain synchrony can be achieved using multibrain stimulation (MBS).

Box 1 **Inferring Causality in Neuroscience**

Dissecting causal from correlational relationships is a central problem in neuroscience. When measuring two variables simultaneously, such as brain activity from two distinct regions, or brain activity and behavior, it is often important to infer whether one variable causes another. Merely observing that one variable precedes another is not sufficient to infer causality: for example, a barometer indicating bad weather is not the cause of a subsequent thunderstorm.

To overcome this problem, two distinct approaches are typically used. A first, interventional approach requires brain stimulation: a wide range of techniques that are used to interfere with brain activity of a given region, and monitor the effects upon a second region or behavior. Alternatively, a second, analytical approach entails the use of algorithms such as Granger causality [12]: brain activity recorded from a given region is used to predict the activity of a second region or various forms of behavior.

While both approaches can undeniably contribute to understanding the neurophysiology of social interactions, we argue that only the interventional approach can provide direct evidence of causality, whereas the analytical one can only indirectly infer it – also considering that when there are conflicting causal models that cannot be teased apart with a particular set of variables, no fancy analysis can overcome the problem. Therefore, when possible, interventional studies should be preferred over analytical ones.

Alt-text: Box 1

## MBS Can Prove Causality

MBS entails the simultaneous stimulation of multiple brains engaged in a social interaction, in order to manipulate interbrain synchrony. This reverses the dominant hyperscanning approach, according to which one manipulates social interaction (independent variable) and measures interbrain synchrony (dependent variable). In MBS, it is interbrain synchrony that is manipulated (as the independent variable), while changes in social interaction are measured (as the dependent variable) (Figure 1A). Hence, using MBS, it is possible to establish whether interbrain synchrony causally modulates social interaction, and eventually confront the mechanistic and epiphenomenal explanations of interbrain synchrony.

Evidence from three different laboratories has shown that this approach is feasible, for example by using transcranial alternating current stimulation simultaneously in two individuals (hyper-tACS; [5., 6., 7.]). One study demonstrated that pairs’ accuracy in establishing interpersonal coordination in a finger-tapping task is augmented when their motor cortices are stimulated with beta band (20 Hz) in-phase currents [6]. Another study has shown that MBS improves learning outcome when the inferior frontal cortices of a student and an instructor are simultaneously stimulated with theta band (6 Hz) in-phase currents [7].

## Future Perspectives for MBS

MBS is an experimental approach still in its infancy. For example, hyper-tACS paradigms have so far addressed a limited number of homologous brain regions, interaction tasks, and signal manipulations [5., 6., 7.]. Relative to the latter, experimenters have mostly delivered constant frequency and amplitude signals, with limited exploration of phase differences (besides perfect in-phase or anti-phase). However, it is well known that neural networks operate in a more complex manner [4], especially in the context of naturalistic social interactions. As the most recent tACS technology permits to (i) control several signal properties; (ii) easily target a number of different (including heterologous) brain regions; and (iii) do this in the context of different tasks, it is clear that this approach has been underexploited. In particular, hyper-tACS could be further sophisticated to induce neural signals simulating the interbrain synchronization observed during naturalistic social interactions.

tACS is by no means the sole way to achieve MBS. In our opinion, at least two viable alternatives have high potential and will be widely used in the future (Figure 1B). First, carefully controlled sensory stimulation could be used to induce and manipulate interbrain synchrony [8]. This approach capitalizes on the crucial evidence that the entraining effects of rhythmic sensory stimuli often outlast sensory stimulation [4,9]. It follows that this property could be exploited to induce coupled neural rhythms in two brains simultaneously, for example presenting two different sensory stimuli (one to each brain), which nonetheless elicit similar neural entrainment. Once such entrainment is reached and the stimuli have stopped, social interaction could be implemented and assessed (notably, following and not during sensory stimulation).

Additionally, more-invasive neurostimulation techniques such as intracerebral electrical stimulation or optogenetics can be used to deliver MBS, especially considering that interbrain synchrony is not unique to humans, but also occurs in other species [10,11]. This approach would lead to a significant gain of spatial specificity with respect to the targeted neural network and cellular type, thus overcoming a well-known limitation of noninvasive transcranial stimulation protocols such as tACS. Of note, one recent study demonstrated that separate cellular populations within the rodent prefrontal cortex, preferentially encoding either one’s own behavior and that of a social partner, differentially contribute to interbrain synchrony [11].

## Combining Hyperscanning with MBS

A final exciting prospect that we wish to highlight is the possibility to combine MBS and hyperscanning. This approach is particularly fruitful because MBS and hyperscanning offer complementary rather than alternative advantages. Specifically, hyperscanning is necessary to identify the social behaviors associated with interbrain synchrony in the context of naturalistic and unrestricted social interactions. Next, MBS should be used to simulate such interbrain synchrony exogenously, and thereby quantitatively measure its effects upon social behavior. Intriguingly, hyperscanning and MBS might be used simultaneously if one wished to assess whether interbrain synchrony has really occurred following MBS.

## Concluding Remarks

Although the use of hyperscanning is central to investigate the neurophysiology of social interactions, MBS offers the only validated empirical approach capable of teasing apart the mechanistic from the epiphenomenal interpretation of interbrain synchrony. Turning the insights achieved through hyperscanning from correlational to causal, MBS is likely to lead to a paradigm shift in social neuroscience. We have described multiple ways how this could be achieved. We forecast that the implementation of MBS, alone or combined with hyperscanning, will yield ground-breaking discoveries in the coming years.
